# Chemical composition of kabuli and desi chickpea (*Cicer arietinum* L.) cultivars grown in Xinjiang, China

**DOI:** 10.1002/fsn3.3056

**Published:** 2022-09-15

**Authors:** Shiqi Xiao, Zhenglei Li, Keqiang Zhou, Yinghua Fu

**Affiliations:** ^1^ Xinjiang Key Laboratory of Biological Resources and Genetic Engineering, College of Life Science and Technology Xinjiang University Urumqi China

**Keywords:** chemical composition, chickpea, flavonoids, isoflavones, UPLC‐QqQ‐MS

## Abstract

Chickpeas are a very important legume crop and have abundant protein, carbohydrate, lipid, fiber, isoflavone, and mineral contents. The chemical compositions of the four chickpea species (Muying‐1, Keying‐1, Desi‐1, Desi‐2) from Xinjiang, China, were analyzed, and 46 different flavonoids in Muying‐1 were detected. The moisture content ranged from 7.64 ± 0.01 to 7.89 ± 0.02 g/100 g, the content of starch in the kabuli chickpeas was greater than that in the desi chickpeas, the total ash content ranged from 2.59 ± 0.05 to 2.69 ± 0.03 g/100 g and the vitamin B_1_ content of the chickpeas ranged from 0.31 to 0.36 mg/100 g. The lipid content ranged from 6.35 to 9.35 g/100 g and the major fatty acids of chickpeas were linoleic, oleic, and palmitic acids. Both kabuli and desi chickpeas have a high content of unsaturated fatty acids (USFAs), Muying‐1 and Desi‐1 contained the highest level of linoleic acid, and Keying‐1 had the highest oleic acid content. The protein level ranged from 19.79 ± 2.89 to 23.38 ± 0.30 g/100 g, and the main amino acids were aspartic acid, glutamic acid, and arginine acid. The four chickpea species had significant amounts of essential amino acids (EAAs). Forty‐six varieties of flavonoids in Muying‐1 were determined by ultra high‐performance liquid chromatography coupled with triple quadrupole mass spectrometry (UPLC‐QqQ‐MS) analysis, and there were higher levels of conjugate flavonoids (55.95%) than free flavonoids (44.05%). Isoflavones were the most abundant flavonoids in Muying‐1, and among the isoflavones, daidzin had the highest content, followed by biochanin A and genistin. Muying‐1 was rich in daidzin, biochanin A, genistin, troxerutin, isorhamnetin, astilbin, L‐epicatechin, astragalin, acacetin, hyperoside, and myricitrin. Information provided in the study will be helpful to further understand the chemical composition of chickpeas and be beneficial to the development of chickpeas.

## Graphical abstract

Chemical compositions such as protein, fat, ash, moisture, starch, vitamin B1 and mineral, as well as amino acids and fatty acids were determined in kabuli and desi chickpea species. Forty‐six types of flavonoids in Muying‐1 chickpea were analyzed with UPLC‐QqQ‐MS method.

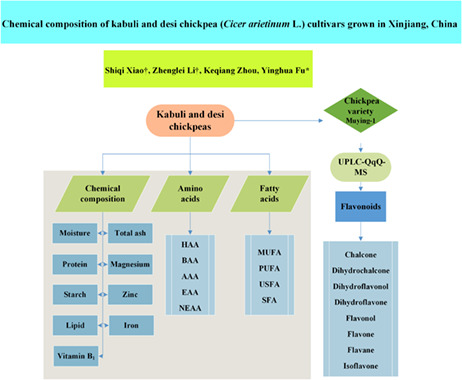

## 1 INTRODUCTION

Chickpea (*Cicer arietinum* L.) is an ancient pulse crop and is the world's third most essential food legume, as it is currently cultivated in over 13.7 million hectares, with an annual production of 14.3 million tons in 2019 (FAOSTAT, 2021). Chickpea is used as a high‐energy and protein source in human diets and is widely consumed because of its high nutritional value (Gao et al., 2015).

Chickpeas mainly come in two varieties, namely, kabuli and desi types. In general, the surface of the kabuli chickpea has a beige coat and it is a large seed without edges, while the desi chickpea has a dark‐colored coat over small and rough seeds (Mohammadi, 2015). Summo et al. (2019a) pointed out that different cultivated chickpea cultivars had different physicochemical, nutritional, and functional properties. For example, brown chickpeas could be used to make vegan burgers or bread and meat extenders, while beige chickpeas had better functional properties and applications in other food products. Both desi and kabuli chickpeas are rich in starch, proteins, carbohydrates, lipids, fibers, flavonoids, and micronutrients such as zinc, iron, and manganese, which are commonly used in the prevention and treatment of chronic diseases such as diabetes and hyperlipidemia.

Legumes are known for their high protein content and chickpea is the cheapest source of protein for the poor people, economically weaker families, and vegetarians (Kaur & Prasad, 2021). Chickpea has potential use as a source of protein, carbohydrates, minerals, and many other important nutrient sources. Chickpea has significant amounts of all of the essential amino acids (EAAs) (Yegrem, 2021). Chickpea protein has advantages of high production volumes, low cost, excellent balance in the composition of essential amino acid, high bioavailability, and low allergenicity compared to soybeans (Boukid, 2021). Wang et al. (2010) found that compared with soybean protein isolates, Chinese kabuli and desi chickpea protein isolates had higher digestibility values.

It has been reported that the content of total starch in chickpea seeds is as high as 40%–60%, approximately 52.5 g/100 g, of which 35% is resistant starch and the remaining 65% is available starch (Aguilera et al., 2009). The total lipid content of chickpea is 4.5–6.0 g/100 g (Boye et al., 2010). The total dietary fiber (DF) content of chickpea is 18–22 g/100 g, and the dietary fiber (DF) concentration is more than twice that of cereal or oilseed bean (Chen et al., 2016). Chickpea is reported to be a good source of iron and it contains a higher level of iron in comparison with other legumes (Abebe et al., 2006).

Chickpeas also contain a large number of biologically active molecules, including steroids, flavonoids, and terpenes, which play an important role in maintaining human health. Chickpea is an important source of flavonoids in human diet. The majority of the flavonoids and phenolics present in chickpea are concentrated mainly in the seed coat and the levels of these compounds are 11, 13, and 31 times (flavonoid, total phenolic, and antioxidant activity, respectively) greater in brown Desi type as compared to creamish Kabuli type (Kaur & Prasad, 2021).

In addition, isoflavones, a large group of plant secondary metabolites belonging to the molecular class of flavonoids, are important components in legumes that exhibit strong antiestrogenic, antioxidative, and antimicrobial activities (Zhao et al., 2009). Isoflavones also presented cancer prevention due to nonhormonal mechanisms, and these compounds suppress angiogenesis, induce apoptosis, and inhibit DNA topoisomerases and cancer cell differentiation (Dulce‐María et al., 2021). The NAD^+^/NADH (nicotinamide adenine dinucleotide) redox imbalance and mitochondrial complex I dysfunction in the pancreas of type 2 diabetes mellitus (T2DM) rats were corrected by chickpea flavonoids (Fu et al., 2022). In naturally occurring glycosidic form, isoflavones are conjugated at the 7′ position and are often esterified with acetyl or malonyl groups at the 6′ position of the sugar residue (Taylor et al., 2005). Konar et al. (2012) reported that chickpeas may serve as an alternative to soy because they are a significant source of isoflavones (3078 ± 372 μg/kg total content) and are also rich in biochanin A. Shang et al. (2019) reported that the yields were 0.55, 1.56, 1.71, 2.40, and 8.35 mg/g for ononin, sissotrin, formononetin, biochanin A, and total flavonoid content, respectively, in chickpea sprouts. The content of total isoflavones in germinated chickpea was about 5 times of that in germinated soybean (Wang et al., 2021).

At present, the cultivated area of chickpea in Mulei County of Xinjiang has exceeded 0.67 million hectares, accounting for 83% of the total planted area of chickpea in China. Muying‐1 chickpea is the main cultivated variety in Xinjiang because of its high yield, good taste, and remarkable economic benefits. Keying‐1, Desi‐1, and Desi‐2 chickpeas are also grown in Mulei County, but Keying‐1 is less stable than Muying‐1, and the two Desi type chickpeas' color, taste, processing performance, and powder yield are inferior to those of Kabuli type chickpeas, so the cultivation of Keying‐1, Desi‐1, and Desi‐2 chickpeas gradually declines. Further insights into the chemical compositions of the four chickpea species in Mulei County of Xinjiang could make their beneficial effects widely known and better support their cultivation and marketing. The purpose of this study was to determine the chemical composition and flavonoids of chickpeas and lay a better foundation of chickpea utilization. With this premise, the mean concentrations of moisture, protein, fat, starch, ash, minerals, and vitamin B_1_ of the four chickpea species Muying‐1, Keying‐1, Desi‐1, and Desi‐2 from Mulei County in Xinjiang were determined. The amino acid composition and fatty acid composition of the four chickpea species were analyzed, and the flavonoids of Muying‐1 chickpea samples were determined by ultra high‐performance liquid chromatography coupled with triple quadrupole mass spectrometry (UPLC‐QqQ‐MS).

## 2 MATERIALS AND METHODS

### 2.1 Materials

**FIGURE 1:**
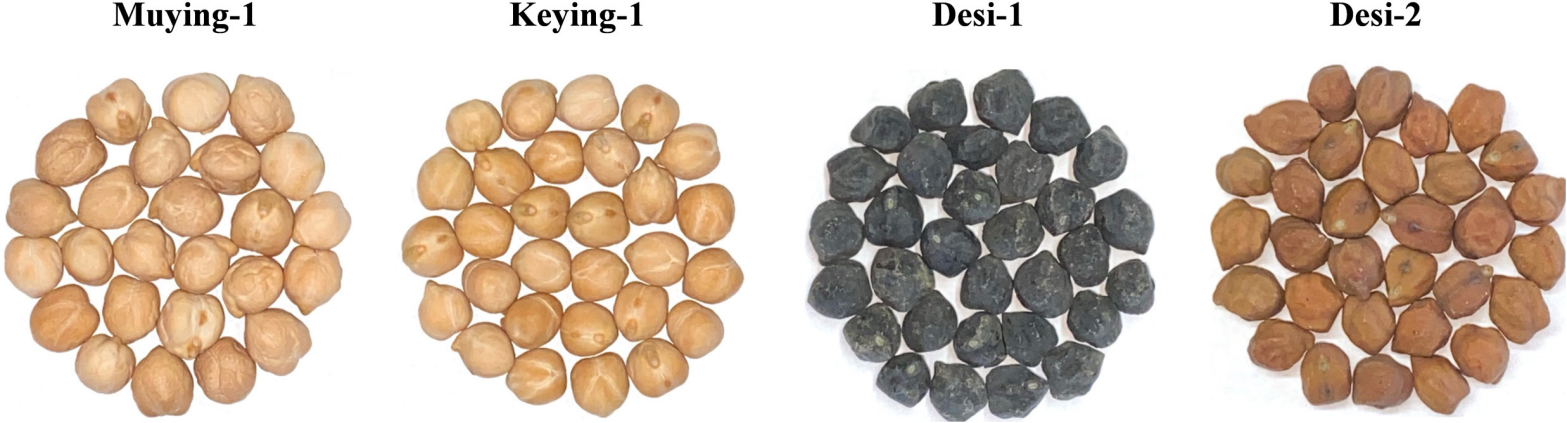
Different varieties of chickpeas from Xinjiang (Muying‐1; Keying‐1; Desi‐1; Desi‐2)

Mature seeds of four commercial chickpea cultivars, namely Muying‐1 (Kabuli type), Keying‐1 (Kabuli type), Desi‐1 (Desi type), and Desi‐2 (Desi type), were collected from Yingge Biotechnology Co., Ltd. (Mulei, China) (Figure 1). The samples were ground to a fine powder (80 mesh) using a mill (FLB‐500A, Philiber Food Machinery Co., Ltd., Shanghai, China) and then stored in an airtight opaque plastic container at 4°C.

### 2.2 Chemical composition analysis

The ash content, vitamin B_1_ content, moisture level, and total crude protein (nitrogen factor of 6.25) were determined according to the AOAC (Association of Official Analytical Chemists) official method (AOAC, 2006). The lipid content was determined by means of a Soxhlet apparatus using petroleum ether as the extracting solvent. The minerals, such as magnesium, zinc, and iron, were determined by inductively coupled plasma atomic emission spectrometry (ICP‐AES). The total starch content was measured using the enzyme hydrolysis method (Rayo‐Mendez et al., 2019).

### 2.3 Amino acid analysis

The amino acids of chickpea flour were determined using an automatic amino acid analyzer (S433D, Sykam, Germany) according to the Chinese Standard (GB 5009.124‐2016) (2016a).

The sample (0.5000 g) was hydrolyzed with 10 ml 6 mol/L HCl in an evacuated test tube for 24 h at 105°C. The hydrolyzed solution was filtered using a membrane with a pore size of 0.22 μm. The mobile phase was a 0.2 mol/L pH 2.2 citric acid sodium‐buffering system. The amino acid composition was expressed as gram (g) of amino acids per 100 grams of chickpea flour and calculated in Eq. The experimental data were from three replications.

$$X=\frac{c\times F\times V\times M}{m\times 10^{9}}\times 100$$

where X represents the amino acid content of the samples, g/100 g; c represents the concentration of the amino acid in the sample, nmol/mL; F represents the sample dilution ratio; V represents the constant volume of the sample after hydrolysis, ml; M represents the molecular weight of each amino acid; m represents the sample weight, g; 10^9^ represents the coefficient of converting the sample content from nanograms (ng) to grams (g); and 100 represents the conversion factor.

### 2.4 Fatty acid analysis

The fatty acid composition of chickpea was determined by gas‐chromatographic mass spectrometry (GC‐MS) analysis of fatty acid methyl esters. Fatty acid preparation and the GC‐MS system and conditions were the same as those described by the Chinese Standard (GB 5009.168‐2016) (2016b). The results are expressed as percentages.

The GC‐MS analysis was performed with an Agilent 7890B/5977A gas chromatograph mass spectrometer system equipped with a DB‐5MS capillary column (30 m long; 0.25 mm i.d.). Helium was utilized as carrier gas at a constant flow rate of 1.0 ml/min. The oven temperature program was as follows: 5 min at 60°C, ramped from 60 to 230°C at 3°C/min, and 5 min at 230°C; then, ramped from 230 to 280°C at 20°C/min, and 3 min at 280°C. The injection mode was splitless, the injector temperature was 280°C, and the ionization voltage was 70 eV.

### 2.5 Flavonoid analysis by UPLC‐QqQ‐MS

Chickpea flour (2.0000 g) was defatted by homogenization with petroleum ether (solid/liquid ratio of 1:10 g/ml) at room temperature for 12 h. Then, the defatted samples were extracted with 70% ethanol (solid/ liquid ratio of 1:20 g/ml) for 4 h at 70°C. Next, the extracts were filtered and concentrated with a rotavapor device. Finally, the crude chickpea flavonoid extract (CCFE) was obtained using vacuum freeze drying. CCFE (0.2000 g) was mixed with 0.8 ml of 80% methanol (MeOH), and the mixture was sonicated for 0.5 h at 4°C to extract the components. The solution was allowed to stand for 60 min at 4°C and then centrifuged for 10 min. The residues were reconstituted in 0.4 ml of 80% (v/v) aqueous MeOH (including an internal standard at 200 ng/mL) and shaken for 5 min. The solution was kept at 4°C for 60 min and then centrifuged for 10 min, and the supernatant was collected and injected into the UPLC‐QqQ‐MS system.

A Waters Acquity UPLC system from Waters Technologies equipped with an AB SCIEX 5500 QQQ‐MS detector was used to carry out the analyses of the extracts. An Acquity UPLC HSS T3 analytical column (1.8 μm, 2.1 mm × 100 mm) was used for separation together with deionized water with 0.1% formic acid (A) and acetonitrile (B) as mobile phases. There was a segmented gradient of 10.0% phase B from 0.00 to 1.00 min, followed by ramping of phase B to 90% from 1.01 to 14.0 min and constant 90% B for 1 min more, then phase B to 10% from 15.01 to 15.1 min and constant 10% B for 3 min. The flow rate was 0.3 ml/min. The injection volume was 6 μl. The column temperature was set at 40°C. The electrospray ionization (ESI positive + negative) source was used in multiple reaction monitoring (MRM) mode at a capillary voltage of 4500 V. This method was used to obtain the TICs (total ion chromatograms) of the samples. All extracted samples were injected into the UPLC‐QqQ‐MS system in triplicate. The MS/MS acquisition parameters (MRM mode) used for the identification of the target phytoestrogens are provided in Table 1.

**TABLE 1:** Multiple reaction monitoring (MRM) acquisition parameters

Number	Compound	Precursor ion (m/z)	Product ion (m/z)	Declustering potential (V)	Collision energy (V)	Cell exit potential (V)
Internal standard	Quercetin D5	306	152.9	−140	−31	−13
1	Silibinin	481.2	124.9	−65	−35	−7
2	Puerarin	415.2	295.1	−56	−31	−16
3	Quercetin	300.8	271.1	−87	−37	−8
4	Genistein	269.1	132.9	−47	−38	−22
5	Apigenin	269	116.9	−75	−46	−6
6	Baicalein	269	139	−74	−44	−16
7	Icariin	677.3	531.3	13	20	8
8	Baicalin	445.1	268.9	−69	−28	−15
9	Rutin	609.1	300	−46	−52	−19
10	Hesperetin	301	163.9	−100	−31	−9
11	Troxerutin	346.3	284.4	27	32	17
12	Hesperidin	609.2	301.1	−42	−34	−18
13	Cianidanol	289	108.9	−98	−31	−12
14	Protocatechualdehyde	136.7	65	−143	−36	−8
15	3,4‐Dihydroxybenzoic acid	152.8	108.9	−85	−19	−6
16	Kaempferol	284.5	93	−170	−43	−5
17	Naringenin	270.9	150.9	−77	−25	−8
18	(‐)‐Epigallocatechin gallate	457.1	169	−92	−24	−10
19	Luteolin	284.7	132.9	−150	−43	−7
20	Myricetin	317	150.9	−80	−32	−8
21	Diosmin	607.1	299.1	−81	−34	−16
22	Dihydromyricetin	319	193.1	−115	−16	−6
23	Taxifolin	303	285	−117	−16	−9
24	Morin	301	150.9	−51	−27	−8
25	Fisetin	285	135	−111	−28	−8
26	Chrysin	253	63	−170	−50	−7
27	Neohesperidin	609.1	301.1	−18	−45	−9
28	Myricitrin	463.1	316.1	−100	−36	−9
29	Isoliquiritigenin	254.9	119	−69	−31	−6
30	Hyperoside	463.1	300	−63	−36	−9
31	Tangeretin	372.8	343.1	23	36	11
32	L‐Epicatechin	290.3	228.2	60	28	13
33	Taxifolin 3‐o‐rhamnoside	449.2	97	−82	−30	−5
34	Phloretin	272.8	167	−93	−22	−5
35	Daidzin	415	253	−58	−23	−8
36	Isorhamnetin	317.8	256.3	25	30	15
37	Genistin	432.2	415.3	38	11	13
38	Biochanin A	283.2	268	−138	−29	−15
39	Glycitin	447.2	285.1	11	21	16
40	Quercitrin	447.3	300	−89	−35	−9
41	Vitexin	431.1	311.1	−83	−30	−17
42	Artemisinin	299	284	−95	−30	−8
43	Glycitein	283.1	240	−106	−35	−7
44	Astragalin	447.1	284.1	−38	−36	−9
45	Acacetin	285	207	155	16	12
46	Procyanidin B_2_	579.3	301.2	30	17	6

### 2.6 Statistical analysis

Data from the different experiments were recorded as the means ± standard deviation (SD) of triplicate measurements and were analyzed by SPSS 26.0 software (SPSS, Inc., Chicago, IL, USA). The results were submitted to analysis of variance (ANOVA) and Duncan's multiple range test for means comparison at (*p* < 0.05) significance was applied.

## 3 RESULTS AND DISCUSSION

### 3.1 Chemical composition of chickpea

In this study, the mean concentrations of moisture, protein, fat, starch, total ash, zinc, iron, magnesium, and vitamin B_1_ of Muying‐1, Keying‐1, Desi‐1, and Desi‐2 were determined. As shown in Table 2, the highest value of moisture content was 7.89 ± 0.02 g/100 g in Desi‐1, and the lowest values were found in Muying‐1 (7.66 ± 0.03 g/100 g) and Desi‐2 (7.64 ± 0.01 g/100 g). There were significant differences in moisture contents between Muying‐1 and Keying‐1, Muying‐1 and Desi‐1, Keying‐1 and Desi‐1, and Desi‐1 and Desi‐2 (*p* < .05), but there was no significant difference between Muying‐1 and Desi2. Importantly, the differences in the moisture content were significant, providing an important parameter to be considered during seed storage.

**TABLE 2:** Chemical composition in four varieties of chickpeas

	Muying‐1	Keying‐1	Desi‐1	Desi‐2
Moisture (g/100 g)	7.66 ± 0.03^c^	7.84 ± 0.01^b^	7.89 ± 0.02^a^	7.64 ± 0.01^c^
Protein (g/100 g)	19.79 ± 2.89^b^	23.38 ± 0.30^a^	22.50 ± 1.31^a^	19.82 ± 0.84^b^
Lipid (g/100 g)	8.39 ± 2.65^a^	9.35 ± 2.00^a^	6.65 ± 1.97^a^	6.35 ± 1.65^a^
Starch (g/100 g)	36.21 ± 1.44^a^	36.22 ± 0.55^a^	27.15 ± 1.19^c^	31.83 ± 3.39^b^
Total ash (g/100 g)	2.66 ± 0.02^a^	2.69 ± 0.03^a^	2.66 ± 0.04^a^	2.59 ± 0.05^b^
Zinc (mg/100 g)	3.61 ± 2.45^a^	4.80 ± 2.63^a^	4.47 ± 1.91^a^	5.33 ± 0.40^a^
Iron (mg/100 g)	7.36 ± 2.94^a^	5.49 ± 1.31^a^	9.72 ± 2.79^a^	6.19 ± 0.90^a^
Magnesium (mg/100 g)	197.73 ± 38.12^a^	224.83 ± 46.36^a^	230.52 ± 47.40^a^	214.87 ± 10.85^a^
Vitamin B_1_ (mg/100 g)	0.36 ± 0.01^a^	0.33 ± 0.01^b^	0.31 ± 0.01^c^	0.33 ± 0.01^b^

*Note*: Data were expressed as mean ± SD (*n* = 3). Values in the same column with different superscript letters represent significant differences between varieties at *p* < .05.

The protein content in chickpeas ranged from 19.79 ± 2.89 (Muying‐1) to 23.38 ± 0.30 g/100 g (Keying‐1). There were significant differences in the protein content between Muying‐1 and Keying‐1 (*p* < .05) and between Desi‐1 and Desi‐2 (*p* < .05). However, there were no significant differences in protein content between Muying‐1 and Desi‐2 or Keying‐1 and Desi‐1. Generally, the crude protein content of the different chickpeas represents 12% to 30% of the seeds, with an average value that is commonly 2–3 times higher than that of cereal grains (Wood & Grusak, 2007). In this study, the average amounts of protein from the Muying‐1, Keying‐1, Desi‐1, and Desi‐2 were higher than that from Merella chickpeas (15.70 g/100 g) (Landi et al., 2021) and other legumes such as common bean and Bambara groundnut accessions (Baptista et al., 2017). Several studies have reported that true protein digestibility, biological value, net protein utilization, and protein efficiency ratio of chickpeas are higher than those of most legumes including soybeans (Dadon et al., 2017; Khan et al., 1979; Liu et al., 2008).

The lipid content of four chickpea species was determined to range from 6.35 ± 1.65 to 9.35 ± 2.00 g/100 g. There was no significant difference in lipid content between Muying‐1, Keying‐1, Desi‐1, and Desi‐2 (*p* ≥ .05). Summo et al. (2019b) reported that considering the seed coat color, beige chickpeas showed the highest lipid content, followed by black chickpeas and brown chickpeas. The lipid content of foods is often responsible for their flavor, which in the case of chickpea may contribute to its “nutty” taste (Yegrem, 2021). On a weight basis, the same amount of lipids provides more than twofold the energy generated by the intake of proteins and carbohydrates (Camargo et al., 2019). Camargo et al. (2019) reported that in this sense, due to its lower lipid content (up to twenty‐one‐fold less) compared to that of soybean, chickpea stands out as a good option in weight management.

The starch content in chickpeas ranged from 27.15 ± 1.19 to 36.22 ± 0.55 g/100 g; the values of Muying‐1 and Keying‐1 were the highest, while that of Desi‐1 was the lowest. A significant difference was found in the starch content among the four chickpea species, and the contents of starch in kabuli chickpeas (Muying‐1, Keying‐1) were greater than those in desi chickpeas (Desi‐1 and Desi‐2) (*p* < .05). These results were similar to an investigation by Miao et al. (2009), which showed that the starch contents of kabuli and desi chickpeas were 37.9% and 29.7%, respectively. Kaur et al. (2019) reported that the total starch content in chickpea genotypes ranged from 26.49% to 39.27% with a mean value of 31.43%.

The significant differences in the ash content of chickpeas have an important role in mineral resources. The Keying‐1 variety had the highest total ash content, while Desi‐2 had the lowest (Table 2). No significant differences were found in the total ash contents among Muying‐1, Keying‐1, and Desi‐1 (*p* ≥ .05), but there was a significant difference in the ash content of Desi‐2 compared with those of the other three chickpeas (*p* < .05). The ash contents in Muying‐1, Keying‐1, Desi‐1, and Desi‐2 were lower than those reported in Merella (3.50 g/100 g) and Alta Valle di Misa (4.0 g/100 g) (CREA, 2020).

The different mineral contents of the chickpeas are shown in Table 2. The zinc, iron, and magnesium contents in Muying‐1, Keying‐1, Desi‐1, and Desi‐2 ranged from 3.61 mg/100 g to 5.33 mg/100 g, from 5.49 mg/100 g to 9.72 mg/100 g, and from 197.73 mg/100 g to 230.52 mg/100 g, respectively. No significant difference was found among the mineral contents of the chickpea strains in this study (*p* ≥ .05). The mineral contents in the four chickpea cultivars were higher than those of desi chickpea cultivars grown in Punjab, Pakistan, previously reported by Zia‐Ul‐Haq et al. (2007). Thavarajah and Thavarajah (2012) reported that Zn and Fe contents in chickpea cultivars ranged from 3.7 mg/100 g to 7.4 mg/100 g and from 4.6 mg/100 g to 6.7 mg/100 g, respectively. These results indicated that chickpea might provide a sufficient amount of minerals to meet the human mineral requirements.

The vitamin B_1_ amounts in Muying‐1, Keying‐1, Desi‐1, and Desi‐2 ranged from 0.31 ± 0.01 to 0.36 ± 0.01 mg/100 g. There were significant differences in vitamin B_1_ contents between Muying‐1 and Keying‐1, Keying‐1 and Desi‐1, and Desi‐1 and Desi‐2 (*p* < .05), but there was no significant difference between Keying‐1 and Desi‐2. Muying‐1 had a significantly higher content of vitamin B_1_ than the other chickpea species.

### 3.2 Amino acid composition analysis

The amino acid composition of the four chickpea species is shown in Table 3. The amino acid contents of the four chickpea species varied from 0.37 to 4.50 g/100 g in Muying‐1, from 0.27 to 4.35 g/100 g in Keying‐1, from 0.23 to 3.85 g/100 g in Desi‐1, and from 0.23 to 3.85 g/100 g in Desi‐2. The contents of total amino acids in kabuli chickpeas (Muying‐1, Keying‐1) were significantly higher than those in desi chickpeas (Desi‐1 and Desi‐2) (*p* < .05). Aspartic acid (Asp) (2.80 g/100 g in Muying‐1 and Keying‐1, 2.45 g/100 g in Desi‐1 and Desi‐2), glutamic acid (Glu) (4.50, 4.35, 3.85, 3.85 g/100 g), and arginine acid (Arg) (2.40, 2.65, 2.40, 2.10 g/100 g) were the most abundant amino acids in chickpeas. No significant differences were found in the Arg content among the four chickpea species, and the contents of Asp and Glu in kabuli chickpeas were greater than those in desi chickpeas (*p* < .05).

**TABLE 3:** Amino acid composition (g/100 g chickpea flour) in four varieties of chickpeas

Amino acid	Sample			
	Muying‐1	Keying‐1	Desi‐1	Desi‐2
Asp	2.80 ± 0.01^a^	2.80 ± 0.14^a^	2.45 ± 0.07^b^	2.45 ± 0.07^b^
Thr*	0.82 ± 0.04^a^	0.80 ± 0.16^a^	0.65 ± 0.04^b^	0.63 ± 0.04^b^
Ser	1.25 ± 0.07^a^	1.15 ± 0.07^b^	0.98 ± 0.03^c^	0.98 ± 0.03^c^
Glu	4.50 ± 0.14^a^	4.35 ± 0.21^a^	3.85 ± 0.07^b^	3.85 ± 0.21^b^
Gly	0.95 ± 0.07^ab^	0.98 ± 0.03^a^	0.84 ± 0.04^b^	0.93 ± 0.01^ab^
Ala	1.00 ± 0.01^a^	0.98 ± 0.04^a^	0.86 ± 0.07^b^	0.90 ± 0.01^ab^
Val*	1.10 ± 0.01^a^	1.05 ± 0.07^a^	1.10 ± 0.14^a^	0.99 ± 0.01^a^
Met*	0.37 ± 0.05^a^	0.27 ± 0.01^b^	0.23 ± 0.04^b^	0.23 ± 0.01^b^
Ile*	0.98 ± 0.04^a^	0.97 ± 0.01^a^	0.92 ± 0.04^a^	0.93 ± 0.04^a^
Leu*	1.65 ± 0.07^a^	1.7 ± 0.14^a^	1.45 ± 0.07^a^	1.55 ± 0.07^a^
Tyr	0.73 ± 0.03^a^	0.81 ± 0.23^a^	0.57 ± 0.06^a^	0.61 ± 0.02^a^
Phe*	1.45 ± 0.07^a^	1.50 ± 0.14^a^	1.30 ± 0.01^ab^	1.20 ± 0.01^b^
His	0.77 ± 0.07^a^	0.79 ± 0.14^a^	0.73 ± 0.13^a^	0.65 ± 0.06^a^
Lys*	1.60 ± 0.01^a^	1.75 ± 0.21^a^	1.40 ± 0.01^a^	1.40 ± 0.14^a^
Arg	2.40 ± 0.28^a^	2.65 ± 0.07^a^	2.40 ± 0.28^a^	2.10 ± 0.14^a^
Pro	1.95 ± 0.78^a^	1.60 ± 0.28^a^	1.10 ± 0.01^a^	1.00 ± 0.01^a^
Total amino acids (TAA)	24.31 ± 0.50^a^	24.13 ± 1.80^a^	20.82 ± 0.18^b^	20.38 ± 0.60^b^
Hydrophobic AA	8.49 ± 0.79^a^	8.06 ± 0.66^ab^	6.95 ± 0.01^b^	6.79 ± 0.04^b^
Bitter AA	7.51 ± 0.01^a^	7.77 ± 0.81^a^	6.74 ± 0.05^a^	6.67 ± 0.16^a^
Aromatic AA	2.18 ± 0.10^a^	2.31 ± 0.37^a^	1.87 ± 0.06^a^	1.81 ± 0.02^a^
Essential AA (EAA)	7.96 ± 0.05^ab^	8.03 ± 0.72^a^	7.04 ± 0.11^ab^	6.92 ± 0.21^b^
Non‐EAA (NEAA)	16.35 ± 0.45^a^	16.10 ± 1.07^a^	13.78 ± 0.29^b^	13.46 ± 0.40^b^
EAA/NEAA (%)	48.67 ± 1.04^a^	49.84 ± 1.15^a^	51.13 ± 1.90^a^	51.37 ± 0.01^a^
EAA/TAA (%)	32.73 ± 0.47^a^	33.26 ± 0.51^a^	33.83 ± 0.83^a^	33.94 ± 0.01^a^

*Note*: Data were expressed as mean ± SD (*n* = 3). Values in the same column with different superscript letters represent significant differences between varieties at *p* < 0.05.

* Essential amino acid.

Essential amino acids (EAAs) are indispensable nutrients for the human body and must be obtained from the diet because they are not synthesized by the human body. As shown in Table 3, chickpea had significant amounts of all of the EAAs. A significant difference in EAA contents was found between Keying‐1 (8.03 g/100 g) and Desi‐2 (6.92 g/100 g) (*p* < .05). The contents of EAA in the four chickpea species were higher than those in field peas (*Pisum sativum* L.) (6.62 g/100 g) and hull‐less barley (3.23 g/100 g) (Bai et al., 2018; Witten et al., 2018). In addition, the amounts of EAAs in Muying‐1 and Keying‐1 chickpeas were also higher than those in Valle Agricola chickpeas (7.12 g/100 g) determined by Landi et al. (2021).

The ratios of EAA to NEAA (nonessential amino acid) in Muying‐1, Keying‐1, Desi‐1, and Desi‐2 were 48.67%, 49.84%, 51.13%, and 51.37%, respectively, which conform to the ideal amino acid pattern. No significant differences were found in the EAA/NEAA of the samples *(p* ≥ 0.05). The ratio of EAA to total AA (EAA/TAA) varied within a narrow range of 32.73%–33.94%. There were no significant differences among the EAA/TAA of the four chickpea species in this study. Malunga et al. (2014) also showed that after processing, the amino acid content in chickpea was found to meet the amino acid profile of the WHO/FAO (World Health Organization/Food and Agriculture Organization) reference protein for 0.5–1 and 1–2 year‐old children. In the case of chickpea flour, fermentation was found to result in better texture properties, aroma, and taste, as well as higher levels of EAAs including methionine, cysteine, phenylalanine, tyrosine, and threonine, compared to unprocessed flour (Angulo‐Bejarano et al., 2008).

The total hydrophobic amino acid (HAA) (alanine, valine, methionine, phenylalanine, isoleucine, leucine, and proline) contents of Muying‐1, Keying‐1, Desi‐1, and Desi‐2 were 8.49 g/100 g, 8.06 g/100 g, 6.95 g/100 g, and 6.79 g/100 g, respectively. Muying‐1 had a significantly higher content of HAA than the other chickpea species. The HAA values of the four chickpea species were higher than those of Forastero cocoa beans (4.4 ± 0.5 mg/g) and *thua nao* (5.69 ± 0.9 g/100 g) (Dajanta et al., 2011; Hinneh et al., 2018).

The contents of bitter amino acids (BAAs) (lysine, tyrosine, valine, isoleucine, phenylalanine, and leucine) in Muying‐1, Keying‐1, Desi‐1, and Desi‐2 were 7.51 g/100 g, 7.77 g/100 g, 6.74 g/100 g, and 6.67 g/100 g, respectively. The aromatic amino acid (AAA) (histidine, tyrosine, and phenylalanine) contents of Muying‐1, Keying‐1, Desi‐1, and Desi‐2 were 2.18 g/100 g, 2.31 g/100 g, 1.87 g/100 g, and 1.81 g/100 g, respectively. No significant differences were found among the BAA contents or AAA contents of the four chickpea species.

### 3.3 Fatty acid composition analysis

The fatty acid composition of the four chickpea species is shown in Table 4. The results showed that oleic and linoleic acids were the dominant unsaturated fatty acids in chickpeas. The oleic acid content of Keying‐1 (35.98%) was significantly higher than those of Muying‐1 (24.81%), Desi‐1 (27.73%), and Desi‐2 (26.32%) (*p* < .05). No significant differences were found in the linoleic acid contents between Muying‐1 and Desi‐1, Muying‐1 and Desi‐2, but there was a significant difference in the linoleic acid content of Keying‐1 compared with other chickpeas (*p* < .05). Muying‐1 (57.75%) and Desi‐2 (58.18%) had higher linoleic acid contents. The fatty acid profile of all chickpea cultivars reveals their lipids are a good source of nutritionally essential linoleic and oleic acids. The nutritional value of linoleic acid is due to its metabolism at the tissue level, which produces hormone‐like prostaglandins. The activity of these prostaglandins includes the lowering of blood pressure and the constriction of smooth muscle (Aurand et al., 1987). Linoleic and linolenic acids are the most important essential fatty acids required for growth, physiological functions, and maintenance (Pugalenthi et al., 2004). Jukanti et al. (2012) also confirmed that chickpea consumption, in particular the black type, could help to enhance the dietary intake of healthy and nutritionally essential fatty acids such as linolenic and linoleic acids. The differences among the strains may be that the maturation and environmental conditions of the chickpeas were different (Deng et al., 2018). Dadon et al. (2017) reported that the fatty acid content of chickpeas is influenced by the genotype (cultivar effect), the environment (i.e., growing seasonal profile, edaphic and climatic factors, pests, diseases), and their possible genotype x environment (G x E) interaction. Gul et al. (2008) reported that chickpea planted in autumn had 35% and 63% oleic acid and linoleic acid, respectively, whereas spring chickpea had oleic acid and linoleic acid of 18% and 47%, respectively.

**TABLE 4:** Fatty acid composition in four varieties of chickpeas

Fatty acids (% in oil)	Muying‐1	Keying‐1	Desi‐1	Desi‐2
Myristic (C14:0)	0.21 ± 0.01^a^	0.16 ± 0.01^c^	0.19 ± 0.01^b^	0.15 ± 0.01^c^
Pentadecanoic (C15:0)	0.07 ± 0.01^a^	0.05 ± 0.01^b^	0.06 ± 0.01^ab^	0.06 ± 0.01^a^
Palmitoleic (16:1)	0.25 ± 0.01^ab^	0.19 ± 0.03^b^	0.28 ± 0.05^a^	0.24 ± 0.02^ab^
Palmitic (C16:0)	12.23 ± 0.02^a^	10.35 ± 0.15^c^	11.99 ± 0.12^a^	11.43 ± 0.05^b^
Heptadecanoic (C17:0)	0.08 ± 0.01^a^	0.05 ± 0.01^b^	0.05 ± 0.01^b^	0.05 ± 0.01^b^
Oleic (C18:1)	24.81 ± 1.60^c^	35.98 ± 0.67^a^	27.73 ± 1.46^b^	26.32 ± 0.41^bc^
Stearic (C18:0)	2.19 ± 0.04^a^	1.90 ± 0.05^b^	1.62 ± 0.08^c^	1.58 ± 0.06^c^
Linoleic (C18:2)	57.75 ± 1.46^ab^	49.30 ± 0.63^c^	55.90 ± 1.15^b^	58.18 ± 0.35^a^
α‐Linolenic (C18:3n3)	0.04 ± 0.01^a^	0.03 ± 0.01^a^	0.04 ± 0.01^a^	0.04 ± 0.01^a^
Arachidic (C20:0)	0.97 ± 0.01^a^	0.82 ± 0.01^bc^	0.87 ± 0.05^b^	0.78 ± 0.01^c^
cis‐11‐Eicosenoic (C20:1)	0.66 ± 0.02^a^	0.61 ± 0.01^ab^	0.64 ± 0.04^a^	0.56 ± 0.05^b^
Henicosanoic (C21:0)	0.05 ± 0.01^b^	0.04 ± 0.01^c^	0.06 ± 0.01^a^	0.05 ± 0.01^b^
Behenic (C22:0)	0.47 ± 0.01^a^	0.40 ± 0.01^b^	0.46 ± 0.05^ab^	0.39 ± 0.01^b^
Tricosanoic (C23:0)	0.06 ± 0.01^a^	0.03 ± 0.01^b^	0.04 ± 0.01^ab^	0.05 ± 0.01^a^
Lignoceric (C24:0)	0.17 ± 0.01^a^	0.09 ± 0.01^b^	0.12 ± 0.01^ab^	0.13 ± 0.01^ab^
SFA	16.51 ± 0.10^a^	13.88 ± 0.22^c^	15.41 ± 0.23^b^	14.67 ± 0.03^bc^
MUFA	25.72 ± 1.58^c^	36.78 ± 0.68^a^	28.66 ± 1.38^b^	27.12 ± 0.39^bc^
PUFA	57.77 ± 1.48^ab^	49.34 ± 0.63^c^	55.94 ± 1.16^b^	58.20 ± 0.37^a^
USFA	83.49 ± 0.10^c^	86.12 ± 0.22^a^	84.59 ± 0.23^b^	85.33 ± 0.03^ab^
USFA/SFA	5.06 ± 0.04^c^	6.21 ± 0.11^a^	5.49 ± 0.10^b^	5.82 ± 0.01^ab^

*Note*: Data were expressed as mean ± SD (*n* = 3). Values in the same column with different superscript letters represent significant differences between varieties at *p* < .05.

The highest values of the unsaturated fatty acid (USFA) content were from Keying‐1 (86.12%) and Desi‐2 (85.33%), and the lowest value was from Muying‐1 (83.49%). The monounsaturated fatty acids (MUFAs) of Muying‐1, Keying‐1, Desi‐1, and Desi‐2 were 25.72%, 36.78%, 28.66%, and 27.12%, respectively. The MUFA content of Keying‐1 was the highest compared to the other three samples (*p* < .05). Summo et al. (2019a) reported that considering the seed coat color, beige chickpeas showed higher concentrations of MUFAs than both brown and black chickpeas. The polyunsaturated fatty acid (PUFA) contents of Muying‐1 Keying‐1, Desi‐1, and Desi‐2 were 57.77%, 49.34%, 55.94%, and 58.20%, respectively; the values of Muying‐1 and Desi‐2 were the highest, while that of Keying‐1 was the lowest. There were significant differences in the PUFA contents between Muying‐1 and Keying‐1, Keying‐1 and Desi‐1, and Keying‐1 and Desi‐2 (*p* < .05), but there was no significant difference between Muying‐1 and Desi‐2. Owing to the high level of PUFAs, chickpeas can be considered nutritionally valuable pulses.

The saturated fatty acid (SFA) contents of Keying‐1, Desi‐1, and Desi‐2 were significantly lower than that of Muying‐1 (*p* < .05). The saturated lipid fraction was represented by palmitic acid, followed by stearic acid in chickpeas. In particular, the palmitic and stearic acid contents accounted for 12.23% and 2.19%, respectively, in Muying‐1 chickpeas, 10.35% and 1.90%, respectively, in Keying‐1 chickpeas, 11.99% and 1.62%, respectively, in Desi‐1 chickpeas, and 11.43% and 1.58%, respectively, in Desi‐2 chickpeas.

Moreover, the USFA/SFA ratio indicated that chickpeas contained more USFA, which has important nutritional and health implications, since the consumption of unsaturated fatty acids is highly recommended to reduce the risk of cardiovascular diseases. There were significant differences in the USFA/SFA ratio between Muying‐1 and Keying‐1, Muying‐1 and Desi‐1, and Keying‐1 and Desi‐1 (*p* < .05), but there was no significant difference between Desi‐1 and Desi‐2. The USFA/SFA ratio of Keying‐1 was the highest compared to the other samples. The fatty acid composition and high amounts of unsaturated fatty acids make chickpea a special legume, suitable for many nutritional applications.

### 3.4 Flavonoids of Muying‐1 chickpea

**FIGURE 2:**
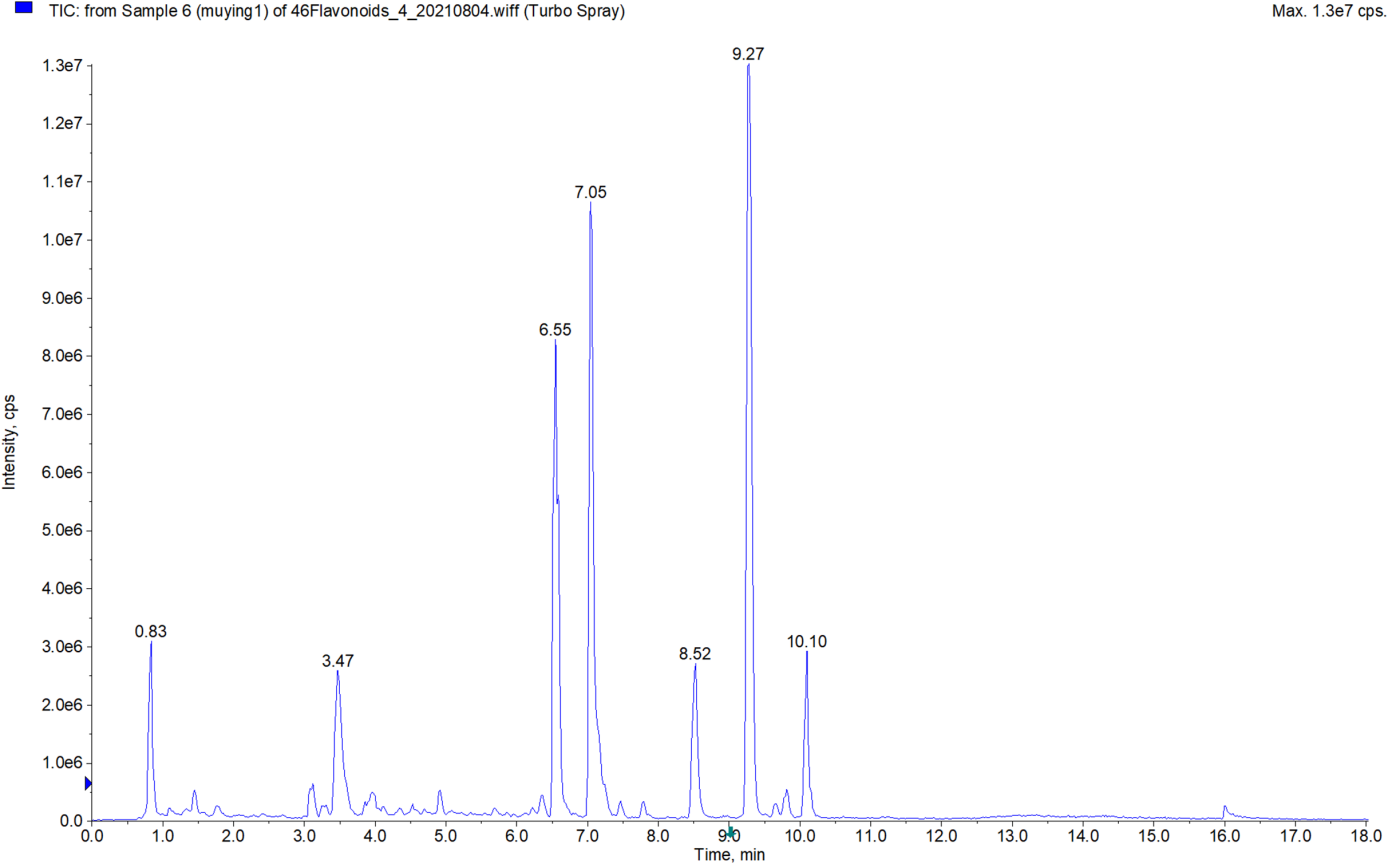
Total ion chromatograms (TICs) that were obtained for the detection and quantification of flavonoids in crude chickpea flavonoid extract (CCFE)

The cultivated area of chickpea in Mulei County of Xinjiang has exceeded 0.67 million hectares, accounting for 83% of the total planted area of chickpea in China. Muying‐1 is the main chickpea variety in Xinjiang because of its high yield, good taste, and remarkable economic benefits. Flavonoids are normal constituents of the human diet and are known for a variety of biological activities. In recent years, flavonoids have been used to improve the oxidative stability of foods (Morelo et al., 2019). Ullah et al. (2019) reported that flavonoids have several beneficial health effects such as anticancer and antiobesity effects and are beneficial against nicotine‐related diseases. Therefore, in the present study the flavonoids in Muying‐1 chickpea were analyzed by UPLC‐QqQ‐MS technology and the results will provide a basis for the subsequent utilization of chickpeas. The TICs for 46 types of flavonoids in Muying‐1 chickpea are shown in Figure 2. In addition, the flavonoids in Muying‐1 crude chickpea flavonoid extract (CCFE) analyzed by UPLC‐QqQ‐MS measurements are reported in Table 5.

**TABLE 5:** The 46 flavonoids of Muying‐1 chickpea using the internal standard method (IS method) by UPLC‐QqQ‐MS (ultra high‐performance liquid chromatography coupled with triple quadrupole mass spectrometry)

Number	Compound	CAS	Molecular formula	Retention time (min)	Flavonoid content (ng/g)
1	Silibinin	22888‐70‐6	C_25_H_22_O_10_	6.85	1.384
2	Puerarin	3681‐99‐0	C_21_H_20_O_10_	3.43	0.563
3	Quercetin	117‐39‐5	C_15_H_10_O_7_	6.99	14.627
4	Genistein	446‐72‐0	C_15_H_10_O_5_	7.13	12.056
5	Apigenin	520‐36‐5	C_15_H_10_O_5_	7.08	8.369
6	Baicalein	491‐67‐8	C_15_H_10_O_5_	7.54	8.544
7	Icariin	489‐32‐7	C_33_H_40_O_15_	6.24	0.254
8	Baicalin	21967‐41‐9	C_21_H_18_O_11_	7.13	6.779
9	Rutin	153‐18‐4	C_27_H_30_O_16_	4.31	74.046
10	Hesperetin	520‐33‐2	C_16_H_14_O_6_	7.37	1.434
11	Troxerutin	7085‐55‐4	C_22_H_34_O_3_	9.92	705.812
12	Hesperidin	520‐26‐3	C_28_H_34_O_15_	5.04	53.149
13	Cianidanol	154‐23‐4	C_15_H_14_O_6_	16.2	8.122
14	Protocatechualdehyde	139‐85‐5	C_7_H_6_O_3_	3.11	2186.378
15	3,4‐Dihydroxybenzoic acid	99‐50‐3	C_7_H_6_O_4_	2.26	121.811
16	Kaempferol	520‐18‐3	C_15_H_10_O_6_	9.3	17.026
17	Naringenin	480‐41‐1	C_15_H_12_O_5_	7.09	15.151
18	(‐)‐Epigallocatechin gallate	989‐51‐5	C_22_H_18_O_11_	3.71	2.167
19	Luteolin	491‐70‐3	C_15_H_10_O_6_	6.31	48.126
20	Myricetin	529‐44‐2	C_15_H_10_O_8_	7.09	6.218
21	Diosmin	520‐27‐4	C_28_H_32_O_15_	4.94	0.656
22	Dihydromyricetin	27200‐12‐0	C_15_H_12_O_8_	3.45	41.169
23	Taxifolin	480‐18‐2	C_15_H_12_O_7_	4.23	64.036
24	Morin	480‐16‐0	C_15_H_10_O_7_	5.91	3.278
25	Fisetin	528‐48‐3	C_15_H_10_O_6_	6.31	47.219
26	Chrysin	480‐40‐0	C_15_H_10_O_4_	8.91	0.438
27	Neohesperidin	13241‐33‐3	C_28_H_34_O_15_	5.03	20.252
28	Myricitrin	17912‐87‐7	C_21_H_20_O_12_	4.47	454.089
29	Isoliquiritigenin	961‐29‐5	C_15_H_12_O_4_	7.78	1.950
30	Hyperoside	482‐36‐0	C_21_H_20_O_12_	4.46	457.233
31	Tangeretin	481‐53‐8	C_20_H_20_O_7_	9.67	5.744
32	L‐Epicatechin	490‐46‐0	C_15_H_14_O_6_	7.23	636.388
33	Taxifolin 3‐o‐rhamnoside	29838‐67‐3	C_21_H_22_O_11_	0.35	653.933
34	Phloretin	60‐82‐2	C_15_H_14_O_5_	7.02	1.326
35	Daidzin	552‐66‐9	C_21_H_20_O_9_	3.95	3193.189
36	Isorhamnetin	480‐19‐3	C_16_H_12_O_7_	8.59	673.082
37	Genistin	529‐59‐9	C_21_H_20_O_10_	10.1	1000.194
38	Biochanin A	491‐80‐5	C_16_H_12_O_5_	9.27	1157.400
39	Glycitin	40246‐10‐4	C_22_H_22_O_10_	4.07	15.759
40	Quercitrin	522‐12‐3	C_21_H_20_O_11_	4.97	2.833
41	Vitexin	3681‐93‐4	C_21_H_20_O_10_	4.38	1.751
42	Artemisinin	491‐54‐3	C_16_H_12_O_6_	9.18	1.778
43	Glycitein	40957‐83‐3	C_16_H_12_O_5_	6.22	12.174
44	Astragalin	480‐10‐4	C_21_H_20_O_11_	4.91	549.015
45	Acacetin	480‐44‐4	C_16_H_12_O_5_	6.24	512.189
46	Procyanidin B_2_	29106‐49‐8	C_30_H_26_O_12_	0.72	49.809
	Total flavonoid				12,848.900
	Total free flavonoid				5659.3911
	Total conjugated flavonoid				7189.5085
	Chalcone				1.950
	Dihydrochalcone				1.326
	Dihydroflavonol				760.522
	Dihydroflavone				89.985
	Flavonol				3006.509
	Flavone				592.597
	Flavane				3004.675
	Isoflavone				5391.335

The total flavonoid concentrations were 12848.9 ng/g for crude chickpea flavonoid extract (CCFE). Daidzin, biochanin A, genistin, troxerutin, isorhamnetin, astilbin, L‐epicatechin, astragalin, acacetin, hyperoside, and myricitrin were the main flavonoids in Muying‐1. The highest content of flavonoids in chickpeas was daidzein, with an average content of 3193.189 ng/g CCFE, accounting for 24.85% of the 46 total flavonoids, and the lowest content of flavonoids was icariin.

Muying‐1 had a conjugate flavonoid concentration of 7189.5085 ng/kg CCFE and a free flavonoid concentration of 5659.3911 ng/kg CCFE. The conjugate flavonoid amount (55.95%) was higher than the amount of free flavonoids (44.05%). Many studies have focused on the hydrolysis of isoflavone glycosides, as isoflavone aglycones possess higher pharmaceutical activity than isoflavone glycosides (Izumi et al., 2000; Kawakami et al., 2005; Kaya et al., 2008). Isoflavone aglycones are absorbed more quickly in humans than their glycosides (Kawakami et al., 2005). However, isoflavone aglycones exist at low concentrations or are absent in leguminous plants. Yeom et al. (2012) determined the optimal pH and temperature for the conversion of genistin to genistein using β‐glucosidase from *Pyrococcus furiosus*, and the hydrolytic activity and kinetic parameters of the enzyme for producing isoflavone glycosides were investigated. Braune and Blaut (2016) reported that the gut microbiota plays a crucial role in the conversion of dietary flavonoids and thereby affects the health‐promoting effects in the human host.

Moreover, several different flavonoid substances including chalcones, dihydrochalcones, dihydroflavonols, dihydroflavones, flavonols, flavones, flavanes, and isoflavones are reported in Table 5. Regarding the flavonoid categories in chickpeas, Muying‐1 showed the highest level of isoflavones (5391.3 ng/kg), with daidzin as the most abundant isoflavone. The content of dihydrochalcone was the lowest, accounting for approximately 0.01% of the total flavonoid content. Seven types of different isoflavones were determined, and the isoflavone (puerarin, genistein, daidzin, genistin, biochanin A, glycitin, glycitein) amounts were 0.563, 12.056, 3193.189, 1000.194, 1157.400, 15.759, and 12.174 ng/g, respectively. According to the literature, the total isoflavone content varied from 153 to 340 mg/100 g of chickpea and from 165 to 336 mg/100 g of soybean (Cantelli et al., 2017; Singh et al., 2017). Dulce‐María et al. (2021) identified six isoflavones (formononetin, biochanin A, and its glycosides) in sprouted black chickpea by HPLC‐UV‐MS and reported the total isoflavone content increased (from 0.31 to 35.72 μg biochanin A/mg of extract). Kaur and Prasad (2021) reported that biochanin A and formononetin were the most abundant isoflavones present in chickpeas. Pérez‐Martín et al. (2017) obtained and evaluated the isoflavone profiles of different types of chickpeas, lentils, and beans and reported that in the three types of legume, the aglycone content found was higher than that of the glucosides.

## 4 CONCLUSIONS

In the present study, the chemical composition of the four chickpea species Muying‐1, Keying‐1, Desi‐1 and Desi‐2 from Mulei, Xinjiang in China was analyzed, and the flavonoids of Muying‐1 were determined by UPLC‐QqQ‐MS. The results demonstrated that the starch and proteins in chickpeas were their main nutritional components. The starch contents of Muying‐1, Keying‐1, Desi‐1, and Desi‐2 were 36.21, 36.22, 27.15, and 31.83 g/100 g, respectively. The protein content ranged from 19.79 to 23.38 g/100 g, and the four chickpea species were found to be rich in aspartic acid, glutamic acid, and arginine acid. The lipid contents in Muying‐1, Keying‐1, Desi‐1, and Desi‐2 were 8.39, 9.35, 6.65, and 6.35 g/100 g, respectively. Chickpea seeds are characterized by a high content of the essential unsaturated fatty acids such as linoleic acid and oleic acid, as well as saturated fatty acids such as palmitic acid and stearic acid. Forty‐six different flavonoids of Muying‐1 chickpea were detected, corresponding to the UPLC‐QqQ‐MS analysis data. The results showed that isoflavones, such as daidzin, biochanin A, genistin, troxerutin, isorhamnetin, astilbin, L‐epicatechin, astragalin, acacetin, hyperoside, and myricitrin, were the main flavonoids in Muying‐1 chickpea. Our findings highlighted the importance of chickpeas in the human diet and indicated that different types of chickpeas vary in nutritional and functional composition. Food processors should be aware of the different components among different chickpea species, which would be helpful to select specific species for better preparation of legume‐based foods.

## CONFLICT OF INTEREST

The authors declare that they have no known competing financial interests or personal relationships that could have appeared to influence the work reported in this paper.
